# ABA-induced active stomatal closure in bulb scales of Lanzhou lily

**DOI:** 10.1080/15592324.2024.2446865

**Published:** 2024-12-28

**Authors:** Lei Gong, Hai-Qing Liu, Ye Hua, Ya-Yun Zhang, Md. Mahadi Hasan

**Affiliations:** a School of Agriculture and Bioengineering, Longdong University, Qingyang, China; b Gansu Key Laboratory of Protection and Utilization for Biological Resources and Ecological Restoration, Qingyang, China; c State Key Laboratory of Herbage Improvement and Grassland Agro-Ecosystems, College of Ecology, Lanzhou University, Lanzhou, Gansu, China

**Keywords:** Hydrogen peroxide, nitric oxide, stomata aperture, water loss, signaling

## Abstract

Abscisic acid (ABA) mediated stomatal closure is a highly effective mode of active stomatal regulation under drought stress. Previous studies on stomatal regulation have primarily focused on the leaves of vascular plants, while research on the stomatal behavior of bulbous plants remains unknown. In addition, ABA-induced stomatal regulation in bulbs has yet to be explored. Therefore, we aim to investigate the ABA-induced active regulation in the bulb of the Lanzhou lily (*Lilium davidii* var. unicolor). The morphological characteristics of epidermal strips were analyzed along with a stomatal aperture assay to investigate the bulb's stomatal response to ABA. Moreover, the mechanism of ABA signaling was explored using treatments with ABA signaling chemicals and corresponding scavengers. This study revealed that stomata are mainly distributed on the upper part and outer surface of the bulb. The guard cells of the lily bulb are inflated, and the stomata have a nearly circular shape with relatively low stomatal density. Exogenous ABA was found to induce varying degrees of stomatal closure in a dose-dependent manner, with significant stomatal aperture reduction observed after treatment with 10 µM ABA. Overall, the study indicated that both hydrogen peroxide (H_2_O_2_) and nitric oxide (NO) are involved in the ABA-induced stomatal closure process, with H_2_O_2_ functioning as an upstream component of NO.

## Introduction

Stomata are essential structures in almost all terrestrial plants, having evolved during the transition from aquatic to terrestrial life. Stomata typically consist of two guard cells surrounding a central pore.^1^ In vascular plants, stomata are generally found on leaves, young branches, flowers, fruits, seeds, and bulbs.^2,3^ The regulation of stomatal opening is a crucial strategy for plants to manage both biotic and abiotic stresses.^4^ On a global scale, stomata form a link between the soil and the atmosphere, making stomatal movement vital for plant survival, the global water balance, and the carbon cycle.^5,6^

ABA plays a significant role in plant responses to various biotic and abiotic stresses, particularly in the process of stomatal closure during drought conditions.^7,8^ In angiosperms, the ABA content in leaves increases rapidly, triggering the expression of numerous related genes to regulate stomatal closure under soil water deficit and reduced atmospheric humidity, thereby mitigating the potentially fatal effects of drought on plants. Recent studies have focused extensively on the molecular mechanisms of ABA signaling, especially on ABA receptors and downstream signaling components, including secondary messengers such as H_2_O_2_ and NO, and ion channels.^9,10^ The evolution of stomatal function in vascular plants has recently been a topic of significant debate.^11^ The one-step model posits that all land plants possessed the ability for active stomatal control via ABA when stomata first evolved.^12,13^ The gradualistic model suggests that stomata of basal vascular plants, ferns and lycopodium are passive in water control and lack the ability to respond to ABA. This significant finding indicates that the change in the stomatal regulation of water balance from passive to active metabolism manner occurred after the divergence of ferns, about 360 million years ago (c. 360 Ma).^14–18^

Recently, most research on stomata has focused on leaves. However, stomata are also present in specific tissue types, such as the sporangia of ferns, nectaries of angiosperms, petals, and bulbs. Research on these specific tissues is limited, and the regulation patterns of stomatal signaling genes may differ between these various tissues.^1,3^ Bulbs are underground modified stems found in many types of plants and have garnered significant attention and research as important nutrient storage organs. Recent studies have shown that some bulbs, such as those of onions and scallions, contain stomata that can respond to exogenous ABA.^19,20^ However, some bulbs, such as those of narcissus species, do not contain stomata, as confirmed by our recent research. The Lanzhou lily (*Lilium davidii* var. unicolor) is a perennial herb with white bulbous underground rhizomes, renowned in China for its rich nutritional and medicinal properties. As a result, it is widely used for both medicinal and dietary purposes.^21,22^

Currently, the response of bulb stomata in Lanzhou lily to the drought hormone ABA has not been reported, and this study is the first to examine the stomatal morphology and response to exogenous ABA in this bulb. Therefore, our aim was to investigate the relationship between the signal molecules H_2_O_2_ and NO in the ABA-induced stomatal closure signaling pathway. We hypothesized that ABA influences stomatal movement, particularly stomatal closure in Lanzhou lily bulbs, through the involvement of hydrogen peroxide (H_2_O_2_) and nitric oxide (NO). Taken together, this experiment is of great significance for improving the theory of stomatal evolution of vascular plants and provides a theoretical basis for breeding excellent drought-resistant varieties of Lanzhou lily and water and fertilizer management.

## Materials and methods

### Plant materials and growth conditions

Lanzhou lily (*Lilium davidii* var. unicolor) was sampled in Chengguan district of Lanzhou city. Healthy, full, non-damaged fresh lily bulbs of the same size with a growth period of 2–3 y were selected as experimental materials. The bulbs were placed in an artificial climate chamber and treated at (20 ± 2℃) for 5 d (16-h/8-h d/night), with the relative humidity kept at 70–80%.

### Observation of stomatal morphology

Peel off the prepared epidermal strips of Lanzhou lily bulb scales at layers 2–4 (from the outside to the inside), cut them into small pieces of 0.5 cm × 0.5 cm, and observe the morphology of stomata on the outer epidermis and inner epidermis under an optical microscope (Olympus CX21, japan), including stomata type, stomatal distribution and stomatal length (SL), stomatal density (SD), stomatal index (SI) and distal axial ratio (ABR). When measuring related stomatal indicators, four fields were randomly selected for each epidermal strip, and five stomata were randomly selected for each field. The experimental statistics were independently repeated each time (*n* = 20). The mean and standard errors were obtained based on three independent replicates. Stomatal length (SL, µm): refers to the length between the junction points of guard cells. Stomatal width (SD, µm): refers to stomatal aperture, is the widest distance of stomata. Stomatal density (SD, mm^−2^): refers to the number of stomata per unit area. The formula is SD (mm^−2^) = N/A, where N represents the number of pores in the visual field, and A represents the area of the visual field. Distal axial ratio (ABR, %): for two-sides stomatal plants, and the distal axial ratio is the ratio of the number of stomata in the outer epidermis to the total number of stomata on the two surfaces of the bulb. The calculation formula is as follows: ABR (%) = Nabaxial/N total × 100%, where Nabaxial represents the stomatal density on the distal axial surface of the blade, and N total represents the total stomatal density on both sides of the blade.

### Stomatal aperture assay

Stomatal aperture assay of epidermal strips was carried out according to the method by Pei et al. (2000).^23^ The outer (abaxial) epidermis of Lanzhou lily bulbs were peeled with forceps, as quickly as possible without crushing the epidermis. The peeled epidermal strips were cut into tiny pieces about 0.5 cm × 0.5 cm. Epidermal strips were incubated in a petri dish containing opening buffer [10 mm 2-Morpholino ethane sulfonic acid (MES), 50 mm KCl, 20 µM CaCl_2_, pH 6.15] and exposed to light for 2 h (PPFD of 100 µmol m^−2^ s^−1^). In order to test the effect of ABA on stomata, a various concentration of ABA was added to the opening buffer with 0.1 µM, 1 µM, 10 µM, and 50 µM for 1 h, respectively. In order to explore the role of several signal substance on ABA-mediated stomatal closure and understand the relationship between them, ABA, H_2_O_2_, NO donor sodium nitro prusside (SNP), 100 Uml^−1^ H_2_O_2_ scavenging-catalase (CAT) and 200 µM NO scavenger 2-(4-carboxyphenyl)-4,4,5,5-tetremethylimidazolinone-1-oxyl 3-oxide (cPTIO) were added to the opening buffer, the peels were incubated in the corresponding inhibitors for 30 min prior to treatments with 10 µM ABA, 100 µM H_2_O_2_, and 100 µM SNP. Stomatal aperture was recorded by optical microscopy (Olympus CX21, japan). The stomatal aperture was statistically analyzed using the Image J software (https://imagej.nih.gov/ij/). When measuring stomatal aperture, four fields were randomly selected for each epidermal strip, and 10 stomata were randomly selected for each field. For each independent replicate, 40 stomatal apertures were measured, and the mean values and standard errors of stomatal aperture were determined by averaging across three replicates for every treatment.

### Measurement of water loss rate of bulb

In order to study the effect of ABA on stomata, we measured the water loss rate of Lanzhou lily bulb. The leathery outer layer was removed, and second to fourth layers of bulb scales were placed in deionized water (MilliQ, Millipore, Billerica, MA, USA) and 50 µM ABA solution as control and treatment, respectively. After 1 h of treatment in light, fetch out the bulb scales and quickly dry them with absorbent paper under light. Weigh them every 30 min for 3 h. Based on three independent replicates per treatment, the mean values and standard errors of the water loss rate were computed.

### Statistical analysis

The experimental data were analyzed by SPSS 22.0 software using ONE-WAY ANOVA DUNCAN’S multiple statistical analysis. *P*-value less than 0.05 was considered as a significant difference, and the presence of distinct letters or asterisks positioned above the columns in the figures signifies noteworthy statistical differences between control and treatment groups. Sigmaplot software (sigmaplot 12.5, Systat) was used to prepare figures.

## Results

### Morphological traits of Lanzhou lily bulbs

**Figure 1.**
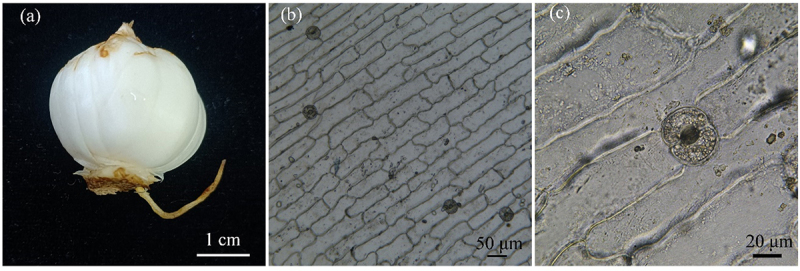
Morphological characteristics of bulb and outer epidermal stomata in Lanzhou lily. (a) Bulb of Lanzhou lily; (b) morphological characteristics of stomata under 10 x objective; (c) morphological characteristics of stomata under 40 x objective.

Through microscopic observation, it was found that stomata were mainly distributed in the upper part of the bulb, there was stomata distribution in both the inner and outer epidermis of the bulbs of Lanzhou lily, and the stomata distribution in the outer epidermis was more than that in the inner epidermis. The guard cells of the bulbs of Lanzhou lily show a reniform shape with equal thick walls, they were parallel-type stomata according to the relationship between stomata and surrounding epidermis cells, and the guard cells contain a large number of chloroplasts with chlorophyll (Figure 1c). Compared with stomatal length, stomatal width is larger, the guard cells of lily bulb are inflated, so the shape of stomata is close to circular, and stomatal density is a little low (Table 1).

**Table 1.** Types and distribution of stomata, stomatal length, width, density, and distal axial ratio in the bulb of Lanzhou lily.

Species	Type and distribution	Stomatal length (SL, µm)	Stomatal width (SW, µm)	stomatal density	Distal axial ratio (ABR, %)
*Lilium davidii* var. unicolor	Reniform double-sided stomata	20.62 ± 2.86	26.18 ± 2.96	3.65 ± 0.53	71.2 ± 9.28

### Stomata of Lanzhou lily bulbs are sensitive to ABA

**Figure 2.**
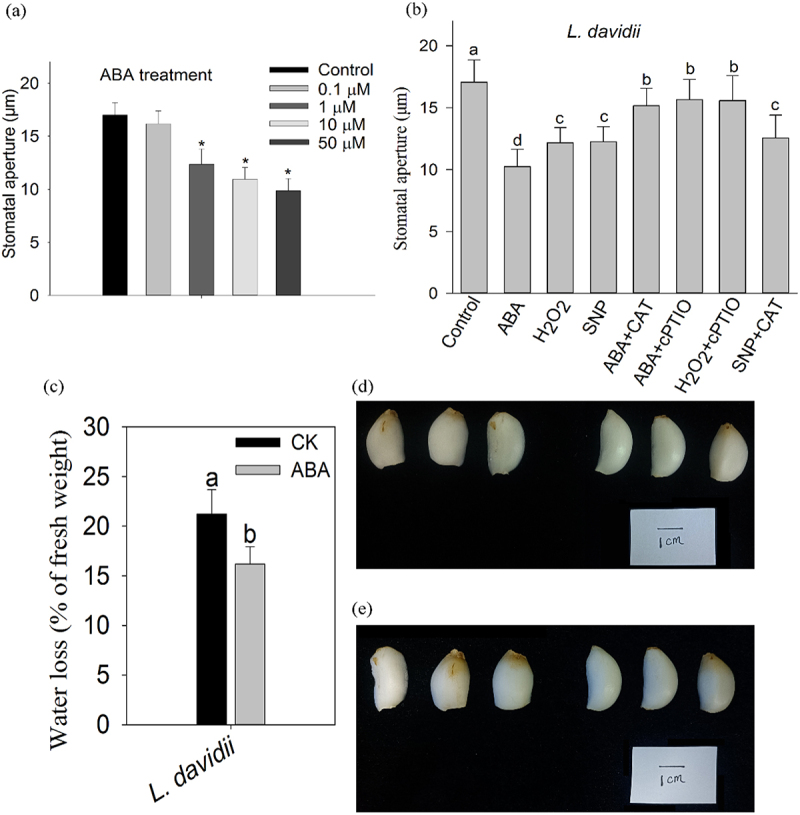
Stomatal responses to ABA and other chemical treatments; (a) stomatal apertures of Lanzhou lily bulbs in responses to different concentrations of ABA. The presented data represents means ± standard error (SE), with a total of 80 stomata counted per treatment and three replicates (*n* = 3). Asterisks are used to indicate statistically significant difference between treatments and the control group (one-way ANOVA, *p* < 0.05); (b) stomatal responses of Lanzhou lily bulbs to various ABA signaling substances. (c) Water loss rate of bulbs after deionized water treatment and ABA treatment in Lanzhou lily; (d) morphology of bulb before treatment; (e) morphology of bulb after water ion water and ABA treatment. The first three scales were treated with deionized water, the last three scales were treated with ABA.

In order to investigate whether the stomata of Lanzhou lily bulbs responded to exogenous ABA, we peeled the outer epidermis of bulbs for stomatal aperture test. After exogenous ABA treatments at different concentrations, the results showed that the stomata of bulb epidermal strips were sensitive to exogenous ABA and there was a concentration gradient effect, and stomatal aperture gradually decreased with the increase in concentration. The stomatal aperture of the bulbs of Lanzhou lily decreased by 35.65% after 10 µM ABA treatment for 1 h. The difference is significant (*p* < 0.05) (Figure 2).

Considering that the concentration of 50 µM ABA may be too high, which has a toxic effect on the epidermal stomata, we selected 10 µM ABA for treatment in the subsequent stomatal aperture experiment. In addition, we measured the water loss rate of isolated bulb scales in the environment, and it was found that after exogenous ABA treatment for 1 h, the water loss rate of isolated scales of Lanzhou lily decreased significantly compared with that of control (*p* < 0.05) (Figure 2). The bulb scales of Lanzhou lily became more crumbled than those in the ABA treatment group after 3 h of deionized water treatment.

### NO plays a role in aba-induced stomatal closure downstream of H_2_O_2_

To further understand how various chemicals influence ABA-induced stomatal closure, we studied the effects of external ABA, H_2_O_2_, NO donor SNP, and their respective scavengers on the stomatal closure in Lanzhou lily’s subterranean bulbs. The experimental results show that ABA and its signaling pathway substances, such as H_2_O_2_, NO, can cause stomatal closure. ABA-induced stomatal closure is inhibited by the peroxide scavenger enzyme catalase (CAT) and the nitric oxide scavenger cPTIO. Similarly, hydrogen peroxide-induced stomatal closure is obstructed by both cPTIO and catalase. However, stomatal closure triggered by nitric oxide is not affected by catalase (Figure 2).

## Discussion

### Function of stomata in the bulb of the Lanzhou lily

Plants growing in different environments can adapt through various mechanisms, such as modifying the morphological characteristics and behavior of their stomata to effectively utilize environmental resources and maintain high levels of physiological activity.^24–26^ In vascular plants, one of the critical functions of stomata is to reduce water loss while maintaining normal photosynthesis.^17,27^ Not all bulbs possess stomata, and our recent study found no stomata on daffodil (*Narcissus tazetta*) bulbs. This raises the question: what is the function of stomata on lily bulbs?

Based on an anatomical examination of the epidermal strips of Lanzhou lily bulbs, it was observed that the guard cells of the lily bulb are swollen, have larger stomatal apertures, and lower stomatal density. More stomata are distributed in the upper part of the bulb and on the outer epidermis (Table 1, Figure 1). Considering that only the guard cells of Lanzhou lily bulbs contain chloroplasts, which facilitate weak photosynthesis by utilizing the little light in the subterranean bulb, we hypothesize that the main role of the stomata is to regulate the opening and closing of stomata and expel water and oxygen produced during respiration and facilitate CO_2_ uptake. Therefore, more stomata are found in the upper part of the bulb to gas exchange in its environment. Due to the higher moisture levels in the soil, the degree of stomatal opening is large, and the bulb does not require a high stomatal density to achieve a high photosynthetic rate in the dim underground light, resulting in a lower stomatal density. Analyzing the epidermal morphological characteristics is valuable for the identification and classification of lily species.

### The stomata in the bulbs of the Lanzhou lily are sensitive to ABA

In vascular plants, stomatal closure has both active and passive modes. In the past, there were disputes about the active regulation of stomatal on leaves. The view of gradualistic evolution suggests that stomatal control of leaf water balance under drought stress was achieved through the accumulation and active regulation of plant hormone ABA in seed plants, while stomatal did not respond to ABA in ferns.^14,15,24^ However, the stomatal response of bulb plants to exogenous ABA is still unclear. In this study, to determine the stomatal responses of stomata to ABA in Lanzhou lily, the stomatal aperture assay was carried out, the results showed exogenous ABA could induce various degrees of stomatal closure in dose-dependent, the stomatal aperture decreased significantly after 10 µM ABA treatment (Figure 2). In addition, we measured the rate of water loss after ABA treatment in Lanzhou lily bulbs and found that the ABA-induced water loss rate of scales decreased significantly with stomata closure (Figure 2). The results indicate that bulb stomata of Lanzhou lily are responsive to exogenous ABA that means the bulb stomata of Lanzhou lily got the ability of active regulation under drought stress.

### Signaling pathway responsible for stomatal responses to ABA

**Figure 3.**
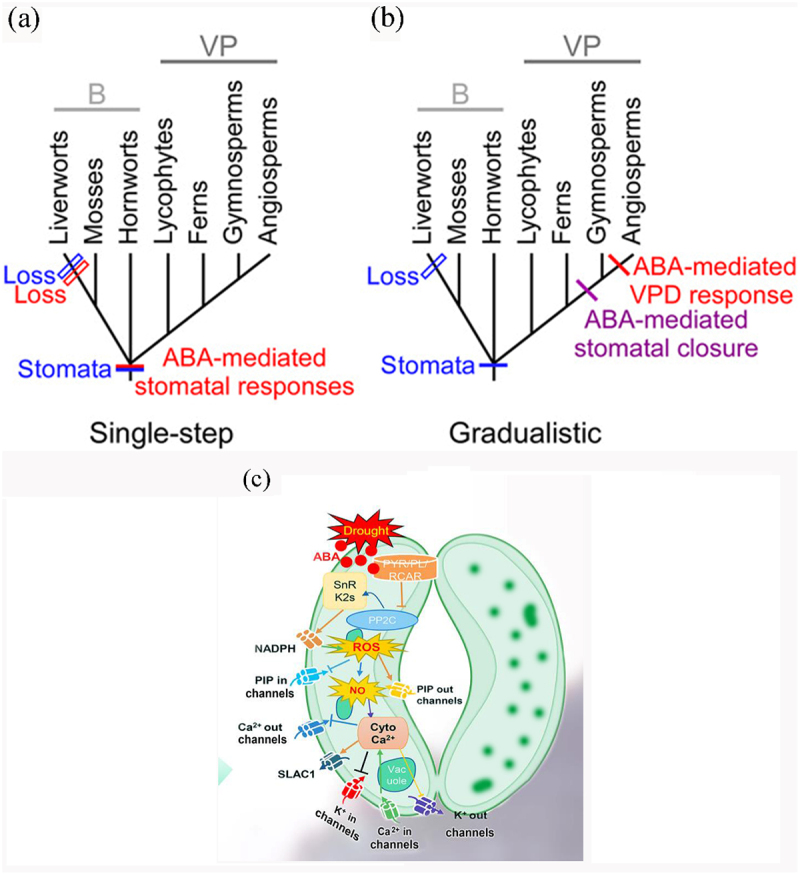
Schematic representations of stomatal evolution and the ABA signaling pathway. Two theories of stomatal function evolution (a) the single-step model; (b) the gradualistic model. Stomatal responses of Lanzhou lily bulbs to exogenous ABA; (c) Model of the ABA pathway, showing that H_2_O_2_ and NO are involved in aba-induced stomatal closure in the bulb scale of Lanzhou lily.

In the past decade, researchers are interested in the stomatal function of earlier-diverged plants. In particular, it has been debated whether or not these plant groups control stomatal movement by active control mechanisms as angiosperms.^12,14,17,28^ The rapid-synthesized ABA in angiosperm leaves under drought stress further induces the synthesis of hydrogen peroxide and nitric oxide, and at the same time, ABA activates various ion channels and the activities of aquaporins in guard cell membrane and finally causes stomatal closure.^4,29,30^ To investigate the signal components of ABA-induced stomatal closure signal transduction pathway in bulb stomata of Lanzhou lily and the relationship between them, the stomatal response was measured by pharmacological experiments. The results showed that both H_2_O_2_ and NO were involved in the process of stomatal closure induced by ABA, and H_2_O_2_ performs function as an upstream component of NO, which is consistent with ABA signaling pathway of stomata in leaves.^31,32^ This suggests that the signaling pathway of ABA-induced stomatal closure may be conserved in different tissues that means both underground bulbs and leaves have developed efficient active stomatal control method that can adapt to drought during the evolution of stomata function, which might explain the wide distribution of Lanzhou lily in arid areas. Previous studies have shown that H_2_O_2_ and NO act in parallel during ABA-induced stomatal closure.^23,33^ Later, studies conducted by He et al. (2005) indicated that endogenous H_2_O_2_-induced NO generation plays an important role in UV-B-induced stomatal closure in *V. faba*.^34^ The signaling pathway is not a simple linear feature, but an intricate signaling web with extensive cross-talk.^35,36^ Sometimes different external stimuli will induce the same signaling pathway and produce the same secondary messenger, such as hydrogen peroxide and nitric oxide (Figure 3).

Therefore, we speculate that the stomata of lily bulb also have the active metabolic regulation of water balance under other stresses.

## Conclusions

In this study, we concluded that stomata were mainly distributed in the upper part of the Lanzhou lily bulbs. The guard cells possess a high concentration of chloroplasts, which are rich in chlorophyll. Compared to the stomatal length, the stomatal aperture is larger, and the shape of the stomata is nearly circular, with relatively low stomatal density. These morphological characteristics may be related to the environment in which the bulb lives. Exogenous ABA was shown to induce varying degrees of stomatal closure in a dose-dependent manner, and 50 µM ABA significantly reduced water loss in Lanzhou lily bulbs. Components of the ABA signaling pathway, such as H_2_O_2_ and NO, can cause stomatal closure, with NO participating in ABA-induced stomatal closure dependent on H_2_O_2_ generation in the bulb stomata of Lanzhou lily. The regulation of stomata is diverse, and more studies on bulb stomata are needed to confirm ABA sensitivity. However, future studies at the molecular level will help to elucidate the internal mechanisms of stomatal closure in bulbs, enhance our understanding of the evolution of stomatal function, and clarify the global distribution patterns of plants.

## References

[1] Sussmilch FC, Schultz J, Hedrich R, Roelfsema MRG. Acquiring control: the evolution of stomatal signalling pathways. Trends Plant Sci. 2019;24(4):342–7. doi:10.1016/j.tplants.2019.01.002. 30797685

[2] Franks PJ, Farquhar GD. The mechanical diversity of stomata and its significance in gas-exchange control. Plant Physiol. 2007;143(1):78–87. doi:10.1104/pp.106.089367. 17114276 PMC1761988

[3] Chen ZH, Chen G, Dai F, Wang YZ, Hills A, Ruan YL, Zhang G, Franks PJ, Nevo E, Blatt MR. Molecular evolution of grass stomata. Trends Plant Sci. 2016;22(2):124–139. doi:10.1016/j.tplants.2016.09.005. 27776931

[4] Zhu JK. Abiotic stress signaling and responses in plants. Cell. 2016;167(2):313–324. doi:10.1016/j.cell.2016.08.029. 27716505 PMC5104190

[5] Lin YS, Medlyn BE, Duursma R, Prentice IC, Wang H. Optimal stomatal behaviour around the world: synthesis of a global stomatal conductance database and scaling from leaf to ecosystem. Agufm. 2014.

[6] Zhou YS, Zhang T, Wang XC, Wu WQ, Xing JJ, Li ZL, Qiao X, Zhang CR, Wang XH, Wang G, et al. A maize epimerase modulates cell wall synthesis and glycosylation during stomatal morphogenesis. Nat Commun. 2023;14(1):4384. doi:10.1038/s41467-023-40013-6. 37474494 PMC10359280

[7] Cathryn JM, Steveninck RFMV. Stomatal closure and inhibition of transpiration induced by (rs)-abscisic acid. Nature. 1969;221(5177):281–282. doi:10.1038/221281a0.

[8] Hewage KAH, Yang JF, Wang D, Hao GF, Yang GF, Zhu JK. Chemical manipulation of abscisic acid signaling: a new approach to abiotic and biotic stress management in agriculture. Adv Sci. 2020;7(18):2001265. doi:10.1002/advs.202001265. PMC7509701 32999840

[9] Zhu SY, Yu XC, Wang XJ, Rui Z, Zhang DP, Fan R-C, Shang Y, Du S-Y, Wang X-F, Wu F-Q, et al. Two calcium-dependent protein kinases, CPK4 and CPK11, regulate abscisic acid signal transduction in Arabidopsis. Plant Cell. 2007;19(10):3019–3036. doi:10.1105/tpc.107.050666. 17921317 PMC2174700

[10] Hauser F, Waadt R, Schroeder JI. Evolution of abscisic acid synthesis and signaling mechanisms. Curr Biol. 2011;21(9):R346–R355. doi:10.1016/j.cub.2011.03.015. 21549957 PMC3119208

[11] Grantz DA, Linscheid BS, Grulke NE. Differential responses of stomatal kinetics and steady-state conductance to abscisic acid in a fern: comparison with a gymnosperm and an angiosperm. New Phytol. 2019;222(4):1883–1892. doi:10.1111/nph.15736. 30740702

[12] Chater C, Kamisugi Y, Movahedi M, Fleming A, Cuming AC, Gray JE, Beerling DJ. Regulatory mechanism controlling stomatal behavior conserved across 400 million years of land plant evolution. Curr Biol. 2011;21(12):1025–1029. doi:10.1016/j.cub.2011.04.032. 21658944

[13] Hõrak H, Kollist H, Merilo E. Fern stomatal responses to ABA and CO2 depend on species and growth conditions. Plant Physiol. 2017;174(2):672–679. doi:10.1104/pp.17.00120. 28351911 PMC5462029

[14] Brodribb TJ, Sam M. Passive origins of stomatal control in vascular plants. Science. 2011;331(6017):582–585. doi:10.1126/science.1197985. 21163966

[15] McAdam SAM, Brodribb TJ. Fern and lycophyte guard cells do not respond to endogenous abscisic acid. Plant Cell. 2012;24(4):1510–1521. doi:10.1105/tpc.112.096404. 22517320 PMC3398560

[16] Duckett JG, Pressel S. The evolution of the stomatal apparatus: intercellular spaces and sporophyte water relations in bryophytes—two ignored dimensions. Philos Trans R Soc Lond B Biol Sci. 2018;373(1739):20160498. doi:10.1098/rstb.2016.0498. 29254963 PMC5745334

[17] Gong L, Liu XD, Zeng YY, Tian XQ, Li YL, Turner NC, Fang XW. Stomatal morphology and physiology explain varied sensitivity to abscisic acid across vascular plant lineages. Plant Physiol. 2021;186(1):782–797. doi:10.1093/plphys/kiab090. 33620497 PMC8154066

[18] Sussmilch FC, Brodribb TJ, McAdam SAM. What are the evolutionary origins of stomatal responses to abscisic acid in land plants? JIPB. 2017;59(4):240–260. doi:10.1111/jipb.12523. 28093875

[19] Jiang ZY, Yu JT, Wang YF, Tan JY. Regulation of stomatal aperture on the outer surface of Allium cepa L. Bot Boreal-Occident Sin. 2011;31(2):315–318.

[20] Peng JL, Ma SM. Exogenous ABA, NO impact on onion bulb on stomatal movement. J Jiangsu Agric Sci. 2011;33(6):262–264.

[21] Li Y, Wang H, Zhang W, Wu H, Wang Z. Evaluation of nutrition components in Lanzhou lily bulb by confocal Raman microscopy. Spectrochim Acta A Mol Biomol Spectrosc. 2021;244:118837. doi:10.1016/j.saa.2020.118837. 32866804 PMC7430252

[22] Kang S, Guo Z, Zhao F, Song L, Lu L, Wang C, Liu Z, Zhao J. Lanzhou lily polysaccharide fragment protects human umbilical vein endothelial cells from radiation-induced DNA double-strand breaks. Hum Exp Toxicol. 2022;41:9603271221140110. doi:10.1177/09603271221140110. 36377570

[23] Pei ZM, Murata Y, Benning G, Thomine S, Klusener B, Allen GJ, Grill E, Schroeder JI. Calcium channels activated by hydrogen peroxide mediate abscisic acid signalling in guard cells. Nature. 2000;406(6797):731–734. doi:10.1038/35021067. 10963598

[24] Cardoso AA, McAdam SAM. Misleading conclusions from exogenous ABA application: a cautionary tale about the evolution of stomatal responses to changes in leaf water status. Plant Signal Behav. 2019;14(7):e1610307–6. doi:10.1080/15592324.2019.1610307. PMC6619974 31032706

[25] Brodribb TJ, McAdam SA, Jordan GJ, Feild TS. Evolution of stomatal responsiveness to CO2 and optimization of water-use efficiency among land plants. New Phytol. 2009;183(3):839–847. doi:10.1111/j.1469-8137.2009.02844.x. 19402882

[26] Casson SA, Hetherington AM. Environmental regulation of stomatal development. Curr Opin Plant Biol. 2010;13(1):90–95. doi:10.1016/j.pbi.2009.08.005. 19781980

[27] Liu H, Song S, Zhang H, Li Y, Niu L, Zhang J, Wang W. Signaling transduction of ABA, ROS, and Ca2+ in plant stomatal closure in response to drought. Int J Mol Sci. 2022;23(23):14824. doi:10.3390/ijms232314824. 36499153 PMC9736234

[28] Cai SG, Chen G, Wang YY, Huang YQ, Marchant DB, Wang YZ, Yang Q, Dai F, Hills A, Franks PJ, et al. Evolutionary conservation of ABA signaling for stomatal closure. Plant Physiol. 2017;174(2):732–747. doi:10.1104/pp.16.01848. 28232585 PMC5462018

[29] Cutler SR, Rodriguez PL, Finkelstein RR, Abrams SR. Abscisic acid: emergence of a core signaling network. Annu Rev Plant Biol. 2010;61(1):651–679. doi:10.1146/annurev-arplant-042809-112122. 20192755

[30] Raghavendra AS, Gonugunta VK, Christmann A, Grill E. ABA perception and signalling. Trends Plant Sci. 2010;15(7):395–401. doi:10.1016/j.tplants.2010.04.006. 20493758

[31] Bright J, Desikan R, Hancock JT, Weir IS, Neill SJ. Aba-induced NO generation and stomatal closure in Arabidopsis are dependent on H2O2 synthesis. Plant J. 2006;45(1):113–122. doi:10.1111/j.1365-313X.2005.02615.x. 16367958

[32] Srivastava N, Gonugunta VK, Puli MR, Raghavendra AS. Nitric oxide production occurs downstream of reactive oxygen species in guard cells during stomatal closure induced by chitosan in abaxial epidermis of Pisum sativum. Planta. 2009;229(4):757–765. doi:10.1007/s00425-008-0855-5. 19084995

[33] Neill SJ, Desikan R, Clarke A, Hancock JT. Nitric oxide is a novel component of abscisic acid signalling in stomatal guard cells. Plant Physiol. 2002;128(1):13–16. doi:10.1104/pp.010707. 11788747 PMC1540198

[34] He JM, Xu H, She XP, Song XG, Zhao WM. The role and interrelationship of hydrogen peroxide and nitric oxide in the UV-B-induced stomatal closure in broad bean. Funct Plant Biol. 2005;32(3):237–247. doi:10.1071/FP04185. 32689127

[35] Hiyama A, Takemiya A, Munemasa S, Okuma E, Sugiyama N, Tada Y, Murata Y, Shimazaki K-I. Blue light and CO2 signals converge to regulate light-induced stomatal opening. Nat Commun. 2017;8(1):1284. doi:10.1038/s41467-017-01237-5. 29101334 PMC5670223

[36] Veith C, Hristova M, Danyal K, Habibovic A, Dustin CM, McDonough JE, Vanaudenaerde BM, Kreuter M, Schneider MA, Kahn N, et al. Profibrotic epithelial tgf-β1 signaling involves NOX4-mitochondria cross talk and redox-mediated activation of the tyrosine kinase FYN. Am J Physiol. 2021;320(3):L356–L367. doi:10.1152/ajplung.00444.2019. PMC8354818 33325804

